# Dysautonomia in Alzheimer's Disease

**DOI:** 10.3390/medicina56070337

**Published:** 2020-07-08

**Authors:** Delia Tulbă, Liviu Cozma, Bogdan Ovidiu Popescu, Eugenia Irene Davidescu

**Affiliations:** 1 Department of Neurology, Colentina Clinical Hospital, Șoseaua Ștefan cel Mare 19–21, 020125 Bucharest, Romania; delia.tulba@umfcd.ro (D.T.); liviu.cozma@drd.umfcd.ro (L.C.); eugenia.davidescu@umfcd.ro (E.I.D.); 2 Colentina—Research and Development Center, Colentina Clinical Hospital, Șoseaua Ștefan cel Mare 19–21, 020125 Bucharest, Romania; 3 Department of Clinical Neurosciences, School of Medicine, Carol Davila University of Medicine and Pharmacy, Bulevardul Eroii Sanitari 8, 050474 Bucharest, Romania; 4 Laboratory of Cell Biology, Neurosciences and Experimental Myology, Victor Babeș National Institute of Pathology, Splaiul Independenței 99–101, 050096 Bucharest, Romania

**Keywords:** Alzheimer's disease, autonomic nervous system, dysautonomia

## Abstract

Alzheimer's disease is the most common neurodegenerative disorder, and its prevalence increases with age. Although there is a large amount of scientific literature focusing on Alzheimer's disease cardinal cognitive features, autonomic nervous system dysfunction remains understudied despite being common in the elderly. In this article, we reviewed the evidence for autonomic nervous system involvement in Alzheimer's disease. We identified four major potential causes for dysautonomia in Alzheimer's disease, out of which two are well-studied (comorbidities and medication) and two are rather hypothetical (Alzheimer's pathology and brain co-pathology). Although there appears to be some evidence linking Alzheimer's disease pathology to autonomic nervous system dysfunction, there is an important gap between two types of studies; histopathologic studies do not address dysautonomia manifestations, whereas clinical studies do not employ histopathologic diagnostic confirmation. Moreover, brain co-pathology is emerging as an important confounding factor. Therefore, we consider the correlation between dysautonomia and Alzheimer's disease to be an open question that needs further study. Nevertheless, given its impact on morbidity and mortality, we emphasize the importance of assessing autonomic dysfunction in patients with Alzheimer clinical syndrome.

## 1. Introduction

Alzheimer's disease (AD) is the most common neurodegenerative disorder worldwide. Its prevalence increases with age, affecting 3% of people aged 65–75 and 32% of people older than 84 years, with further rise expected due to the “baby boomer” effect [1]. AD is characterized by an impairment in multiple cognitive domains that progresses towards dementia, typically with early changes in episodic memory as a consequence of entorhinal cortex damage [2]. The neuropathological hallmarks of AD are cerebral extracellular amyloid plaques embodying amyloid-β and intracellular neurofibrillary tangles comprising hyperphosphorylated tau protein [2,3]. Synaptic degeneration and neuronal death lead to brain atrophy that preferentially involves certain regions [2]. Nevertheless, AD etiopathogenesis is far from being elucidated, and new insights into its mechanisms are expected.

Autonomic nervous system (ANS) mediates homeostasis by controlling several visceral systems and providing specific responses (i.e., autonomic behaviors) that accompany and adjust in relation to physical efforts and mental activities (e.g., emotions, cognitive challenge) [4]. Damage to the ANS that impairs function beyond compensatory mechanisms leads to dysautonomia [4]. The spectrum of dysautonomia manifestations ranges from asymptomatic (detectable only on clinical examination or autonomic testing) to disabling symptoms (orthostatic hypotension, syncope) [4]. Moreover, inappropriate sympathovagal balance and depressed heart interval variability have been recognized as independent risk factors for ventricular arrhythmias, sudden death, and other cardiovascular events [4,5].

Patients with AD have high mortality rates [6], mostly due to events closely related to ANS dysfunction such as bronchopneumonia, myocardial infarction, and cardiac failure [7,8]. Since patients with AD are generally older and have many comorbidities, it is plausible that even slight autonomic dysfunction superimposed on cardiac comorbidities might lead to worse outcomes [5]. Therefore, it is important to thoroughly address autonomic dysfunction in AD patients [9].

## 2. Dysautonomia in Alzheimer's Disease

Dysautonomia frequently occurs in neurodegenerative disorders. Among these, α-synucleinopathies (i.e., multiple system atrophy (MSA), dementia with Lewy bodies (DLB), Parkinson's disease (PD), and Parkinson's disease dementia (PDD)) are commonly associated with moderate to severe autonomic failure (as opposed to autonomic hyperactivity, which usually occurs in acute settings such as stroke) [4]. Nonetheless, in AD, autonomic dysfunction has not been properly characterized and much controversy has arisen, oscillating between denying its existence and considering it as a cardinal feature [10].

Over the past 30 years, evidence that ANS dysfunction develops in patients with AD and worsens with advanced disease has emerged [11]. Moreover, it might occur before the onset of the clinical symptoms of dementia [12]. Patients with mild cognitive impairment (i.e., cognitive impairment that does not interfere with independence in everyday activities) seem to have prominent dysautonomia compared with controls, unevenly distributed among the autonomic divisions, with significant parasympathetic dysfunction [5,13]. On the contrary, another study found only mild to moderate clinical signs of dysautonomia in AD patients, with a prevalence of 66% [10]. The most frequent were orthostatic hypotension (34%); constipation (17%); urinary incontinence (14%); syncope (7%); as well as hypohidrosis, urinary urgency, and diarrhea (each with a prevalence of 3.5%) [10]. Affoo et al. found that dysphagia occurs in 84–93% of AD patients and affects both oral and pharyngeal stages, occasionally in early phases of the disease [14]. They assumed that functional changes in the cortical swallowing network might be responsible for dysphagia and found a direct correlation between its severity and that of AD [14]. However, some authors question the link between dysautonomia and some of these manifestations. For instance, urinary incontinence (so-called pseudoincontinence) might be the result of visual-spatial agnosia that impedes the patient to find the way to the toilet, whereas orthostatic hypotension could be directly related to the process of ageing [10] and is associated with the risk of dementia [15].

In AD, in addition to the impaired autonomic homeostasis of physical efforts, inappropriate autonomic responses are also elicited by cognitive challenge and strong emotions, pointing to a defective connection between mental status and autonomic reply [4,9]. Moreover, altered autonomic acute pain responses (i.e., coherent adaptive responses to noxious stimuli, such as sympathetic activation with increased heart rate) signal impaired pain processing, possibly due to disconnection between behavioral and autonomic responses [11]. Compared to controls, patients with AD have increased pain behaviors and reactivity with reduced autonomic measures [11].

**Figure 1:**
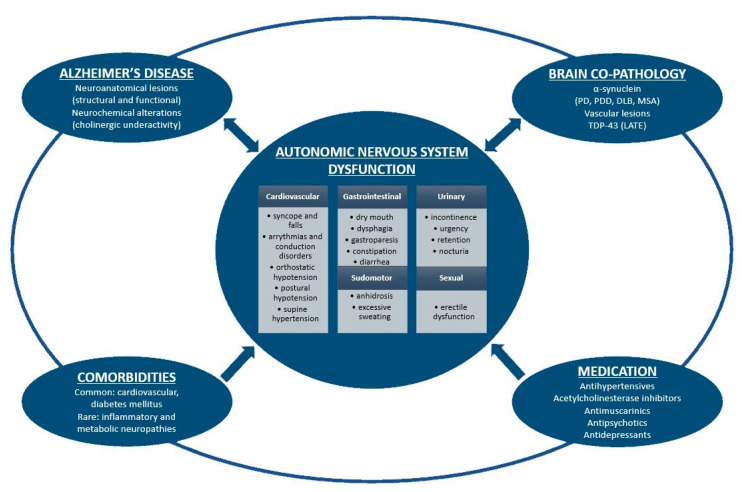
Schematic representation of autonomic nervous system dysfunction in Alzheimer's disease. Alzheimer's disease pathology, brain co-pathologies, comorbidities and medication are intimately interrelated and could all induce autonomic symptoms. On the other hand, dysautonomia might enhance histopathological brain burden in Alzheimer's disease and other proteinopathies. PD: Parkinson's disease; PDD: Parkinson's disease dementia; DLB: dementia with Lewy bodies; MSA: multiple system atrophy; LATE: limbic-predominant age-related TDP-43 encephalopathy.

In an attempt to explain autonomic dysfunction manifestations in patients with AD, we have identified four potential causes: AD pathology involving central and peripheral autonomic structures, brain co-pathologies, comorbidities, and medication (Figure 1).

## 3. Dysautonomia as a Result of Alzheimer's Disease Pathology

ANS dysfunction supposedly arises in AD mainly as a result of central autonomic network impairment by neuroanatomical lesions (structural and functional) and/or neurochemical changes. Central autonomic regions are widely and intimately interconnected, exerting tonic, reflex, and adaptive control over autonomic functions and regulating cognitive, behavioral, and endocrine responses [4].

In AD, the gradual deposition of hyperphosphorylated tau protein occurs in selective vulnerable brain regions in a predictable pattern of distribution and sequence, leading to intra and interneuronal damage [4,16]. Accordingly, Heiko Braak settled a staging system of AD-associated neurofibrillary pathology, identifying six stages (I–VI) of disease process corresponding to three phases (initial: asymptomatic; intermediate: incipient disease; and late: advanced disease) (originally described in 1991 and revised in 2005) [16]. Various structures pertaining to the central autonomic network are prone to neurofibrillary degeneration in different stages of the disease [13].

There is compelling neuroanatomical and electrophysiological evidence to indicate that the ventromedial frontal cortex modulates autonomic responses through direct projections to the subcortical autonomic centers [9]. Among the cortical regions involved in the neurodegenerative process in AD, the autonomic-related ones—namely, Brodmann's area 25, the posterior orbitofrontal cortex, and the anterior insula—are affected progressively from stage III to IV and reach maximal severity in the final stages, comparable to that of the temporal cortices (excluding the entorhinal cortex and the temporal pole), when they overcome the histopathologic strain of any associative areas of the frontal, parietal, and occipital lobes [4,9]. However, the neuronal layers of the ventromedial frontal cortex are not uniformly involved, with layers V and III being the most severely affected. This selective distribution of neurofibrillary tangles leads to the disruption of direct cortico-autonomic connections, with possible contribution to behavioral changes, emotional disturbance, and dysautonomia in AD [4,5,9].

Although less studied in AD, autonomic-related subcortical structures seem to be thoroughly involved in the neurodegeneration process. The amygdala shows significant neurodegenerative changes from initial phases (stages II–III), suffering severe volumetric atrophy throughout the disease, with a neuronal loss of up to 50% [4]. Hypothalamic nuclei are not concurrently or evenly impaired in AD. The lateral nuclei (tuberomammillary, lateral tuberal) are progressively involved from stage IV and severely affected at stage VI, whereas the other regions are later and less extensively damaged (the supraoptic and paraventricular nuclei are followed in severity by the mediobasal and anterior hypothalamus) [4,17]. The anterior, mediodorsal, and laterodorsal thalamic nuclei start showing changes in stages I–II, whilst the anteroventral, paraventricular, and reuniens nuclei display neurofibrillary pathology at stage IV and peak at stage VI [4]. In basal ganglia, the ventral part of the striatum (nucleus accumbens and olfactory tubercle) is earlier and more broadly affected than the dorsal part (stage III–IV versus stage V–VI), whereas the globus pallidus is completely spared [4,18]. Interestingly, the cerebellum also appears to suffer neurodegenerative changes (amyloid plaques, not neurofibrillary tangles) in AD, particularly in the molecular and granular layers [19,20]. Moreover, compared to the cerebellar hemispheres, the vermis undergoes atrophy [4].

Throughout the disease course, the brainstem also becomes involved in the neurodegeneration process. The neurofibrillary pathology is mainly distributed rostro-dorsally and is heterogeneously expressed during different stages, both in the autonomic nuclei and (cell bodies of) preganglionic parasympathetic neurons [4]. Significant neuronal loss has been described in the Edinger–Westphal nucleus and the dorsal vagal motor nucleus, whilst the nucleus solitarius and nucleus ambiguus are affected to various extents [4,21]. It is noteworthy that higher order processing autonomic nuclei residing in the brainstem also exhibit neurodegenerative changes. Among these, there are several reticular formation nuclei (involved in cardiovascular and respiratory control, swallowing, defecation, and micturition), periaqueductal gray, pontine parabrachial nuclear complex, and intermediate reticular zone of the medulla (the last two encompassing relay stations within the central autonomic regulatory feedback system) [4]. The pathological changes in the pontine parabrachial nuclear complex and intermediate reticular zone parallel those of cortical neurodegeneration (i.e., progressive pathology starting in stages I–II, prominent in stages III–IV, and severe in stages V–VI) [4]. Provided that the brainstem and subcortical nuclei, such as the locus coeruleus, nucleus raphes dorsalis, and magnocellular nuclei, of the basal forebrain occasionally become involved even earlier than the cortical regions [16,22], it has been hypothesized that dysautonomia might be present in the preclinical stage of AD [12,13].

Regarding the spinal cord, inconsistent and sparse information exists on AD pathology at this level. Few tangles were identified in the central region and lateral horns of spinal cord (origin of sympathetic preganglionic fibers), occasionally also involving anterior and posterior grey columns [4]. Nevertheless, it seems that the spinal cord is remotely affected in AD without significant clinical impact [4].

To sum up, in AD the neurodegenerative process affects almost all the structures relating to the central autonomic network in different stages of the disease and to various extents [4,5].

However, since functional alterations probably precede structural atrophy, the examination of cerebral functional connectivity might have an advantage over brain morphometry, especially in early stages of the disease [23]. Patients with incipient disease have impaired hippocampal connectivity to the medial prefrontal cortex, ventral anterior and posterior cingulate cortex, and right superior and middle temporal gyrus, worsening as the disease advances [23]. Nevertheless, functional and structural changes should be regarded as two facets of the same process. For instance, in mild cognitive impairment, the basal nucleus of Meynert (the main source of cholinergic innervation of the cortex) undergoes decreased functional connectivity to the left insula and claustrum (which integrate information from various brain regions through their reciprocal projections to neocortex, limbic, and paralimbic regions) as well as neuronal loss with subsequent volume reduction as a result of β-amyloid deposition, neurofibrillary tangles formation, and impaired trophic support [23,24]. However, enhanced connectivity between different structures has also been described, possibly as a mechanism of functional reallocation, to compensate for cognitive decline [11]. One example is the increased connectivity between temporal limbic network and a cluster in the ventromedial prefrontal cortex in patients with behavioral over-responsiveness to pain, possibly extending to other negative emotional traits [11].

In addition to structural and functional changes affecting the central autonomic network, neurochemical alterations also contribute to the autonomic dysregulation in AD [12]. The cholinergic system is mostly affected, with an increase in the insular acetylcholinesterase activity and a decline in the cortical choline acetyltransferase, with ensuing cholinergic synaptic transmission deficiency [5,10,24]. In patients with mild cognitive impairment and early AD, it has been hypothesized that the cholinergic deficiency is not due to basal forebrain cholinergic cell death but is rather the result of the loss of synaptic contacts within the cortical projection regions, as reflected by the shrinkage of cholinergic neurons [24]. The cholinergic-vascular hypothesis assumes that the reduction in cerebral blood flow is the result of the massive loss of cortical perivascular cholinergic nerve terminals [12]. The cholinergic underactivity alters both sympathetic and parasympathetic functions and is critical for memory impairment [12,13,24]. On the other hand, sympathetic hyperactivity also seems to occur in AD, also correlating with poorer cognitive performance [12]. In these patients, the basal plasmatic norepinephrine levels are higher, with enhanced basal sympathoneural activity and cardiovascular responsiveness to sympathoneural stimulation [12]. Lymphocyte G-protein coupled receptor kinase 2 (GRK2) protein, a biomarker of sympathetic dysfunction, is highly expressed in patients with AD, correlating with the severity of cognitive impairment [12].

Although less studied, the involvement of the peripheral nervous system in AD seems to play a role in autonomic dysregulation [5]. In these patients, depressed baroreflex sensitivity and decreased heart rate variability (which correlates with blood levels of acetylcholinesterase activity) have been documented, indicating damage to the peripheral autonomic system in addition to the central autonomic network [12,25]. This issue needs to be addressed, since it reflects an autonomic imbalance (i.e., reduction in parasympathetic activity and increase in sympathetic tone) that might precipitate and aggravate ischemic heart disease [25]. Occasionally, in patients with AD, the dopamine β-hydroxylase-immunostaining of nerve cells within the pineal gland identifies abnormal swollen immunoreactive fibers similar to the neuritic abnormalities that arise in the hippocampus of these patients [26]. Since the pineal gland embodies a plexus of noradrenergic axons originating in the superior cervical ganglion, this finding might prove that the peripheral noradrenergic system is also damaged in AD [26].

## 4. Dysautonomia as a Result of Brain Co-Pathologies

When talking about autonomic dysfunction in AD, it is important to establish the various nuances implied. We have summarized the evidence for AD pathology in the areas involved in central autonomic regulation, but the mere existence of these findings does not inherently equate to the emergence of equivalent autonomic symptoms. In order to state a causal relationship, there must be a clear association between these histopathologic changes and the symptoms of autonomic dysfunction. Unfortunately, most histopathologic studies did not address this question and only focused on the link between cognitive dysfunction and the AD burden, whilst studies trying to prove the existence of ANS dysfunction did not employ histopathologic confirmation.

The new National Institute on Aging-Alzheimer's Association (NIA-AA) Research Criteria for the diagnosis of AD make a clear distinction between AD as a disease with specific histopathologic changes and AD as a clinical syndrome [27]. They propose a framework with indirect markers of β-amyloid and phosphorylated tau pathology to diagnose AD in vivo that is easy to use in a research setting and can be extended to clinical practice in situations of diagnostic uncertainty [27]. Since they are indirect markers, they do not have a 100% sensibility and/or specificity for AD [27]. However, they are much better than clinical symptoms for predicting AD histopathologic changes [27].

Previous diagnostic criteria are designed for use in clinical settings, and for this reason they focus on clinical symptoms related to cognitive dysfunction. Therefore, they only define a dementia syndrome thought to best describe cognitive impairment due to AD. However, these symptoms are far from having great sensibility and specificity, with reports of clinical misdiagnosis of 10–30% when verified by histopathologic examination [27,28]. For this reason, the new NIA-AA Research Criteria recommend using the term “Alzheimer clinical syndrome” when the diagnosis is based on clinical features and the markers of AD pathology are not used [27]. Moreover, AD pathology can lead to atypical presentations that might be misdiagnosed in routine clinical practice. Since they do not have a clear clinical diagnosis of AD, these patients would also be excluded from a study trying to link autonomic symptoms with AD pathology. For instance, one recent study found that 75% of patients with clinical criteria had a high AD burden, but from those with a high AD burden only 59% fulfilled the criteria for a clinical diagnosis of AD [29].

The new research criteria also state that evidence of AD pathology does not necessarily mean that clinical symptoms arise as a consequence of these histopathologic changes [27]. Although it seems reasonable to assume that symptoms are at least partly due to AD pathology, it is currently difficult to establish the impact of co-pathology. Many histopathologic studies have shown that most patients do not have isolated AD pathology, and co-pathology is rather the rule than the exception [29,30,31]. The degree of co-pathology can vary from none or minimal to severe additional burden, with clinical implications possibly ranging from no supplementary symptoms to markedly overlapped phenotypes. For this reason, in the NIA-AA Research Framework it is recommended to formulate a diagnosis of AD with mild cognitive impairment/dementia instead of mild cognitive impairment/dementia due to AD [27].

The best studied co-pathology is the one shared by AD and Lewy body disorders (LBD), and attention has been drawn to the importance of studying how abnormal proteins in these diseases interact with and influence each other [32]. Although autonomic dysfunction is well-known in all disorders within the Lewy body spectrum (Parkinson's disease, Parkinson's disease dementia, and dementia with Lewy bodies), it was not addressed in studies assessing co-pathology, which focused mostly on cognitive and motor symptoms. In a large study of patients who were clinically and histopathologically diagnosed with LBD, AD pathology was present in 77% of them [33]. It was a predictor for shorter intervals of time from the onset of motor symptoms to that of dementia and shorter survival times [33]. Higher levels of AD burden predicted a phenotype of DLB as opposed to PDD [33]. Moreover, 19 of 98 patients with DLB never developed symptoms of parkinsonism, and all of these had an intermediate to high AD burden [33].

In another study, pure AD and pure LBD pathology were found in only a minority of cases, and most AD patients were associated with either α-synuclein or TDP-43 co-pathology [29]. AD pathology was reported in 38% of MSA cases, 50% of brainstem LBD, 57% of limbic LBD, and 80% of neocortical LBD, and it was significantly more widespread in the neocortical LBD group [29]. There was no significant difference in the speed of cognitive deterioration between patients with pure AD and AD with co-pathology, but the presence of AD pathology in those that had LBD led to faster cognitive impairment [29]. Others reported that some patients initially diagnosed with DLB had enough AD pathology to be classified as AD as well, historically known as the Lewy body variant of AD [34].

While most studies focused on the histopathologic coexistence of AD and LBD changes, there is also an increasing awareness of the importance of TDP-43 pathology in patients with cognitive impairment, leading to the recent description of limbic-predominant age-related TDP-43 encephalopathy (LATE) [35]. In one study, although TDP-43 pathology was found to be very common in AD, it was concluded that clinical presentation was related to the pathological subtype of AD and not to the TDP-43 burden [36]. However, the evidence for cognitive impairment in the so-called hippocampal sclerosis of ageing and its relation to TDP-43 deposition was thoroughly reviewed in the characterization of LATE [35]. There are no data concerning autonomic dysfunction in LATE, possibly because dysautonomia is not a noticeable feature or might altogether be absent (an argument that can be extended to AD as well). However, many patients with TDP-43-related dementia might be clinically diagnosed with AD, and studies trying to link autonomic dysfunction to AD could underestimate its prevalence.

There is a significant amount of literature on the impact of classical vascular risk factors, such as arterial hypertension and diabetes mellitus, in developing AD. However, these factors might only lead to vascular lesions that contribute to an existing or non-existing AD pathology. Without histopathologic examination, these risk factors are only linked to an Alzheimer clinical syndrome and not to AD. In one study, patients with autonomic dysfunction, such as orthostatic hypotension and various dementias (AD, LBD, and others), had more histopathologic vascular-related changes [37]. Dysautonomia, either associated with a certain type of dementia or unrelated, could therefore lead to worse cognitive impairment by additional vascular burden. It might accelerate cognitive decline by hypotension-induced cerebral hypoperfusion, presumably triggering proinflammatory responses, oxidative stress, and β-amyloid deposition with synaptotoxic effects [13]. This highlights the importance of effectively treating autonomic symptoms in dementia, irrespective of their cause.

In a study of 512 subjects with a clinical diagnosis of AD, only 41% of them had individual neuropathologic changes of AD, whilst almost 12% were considered to be related to TDP-43 pathology and 11% to LBD [30]. More than 5% were also attributed to each of the following: macroscopic infarcts, hippocampal sclerosis, cerebral amyloid angiopathy, atherosclerosis, and arteriosclerosis [30]. Notably, over 80% of cases had mixed pathology [30].

Taking all these into consideration, it is clearly difficult to establish if autonomic dysfunction in AD is a consequence of AD pathology, and we consider this an open question. Confounding factors might be related to the existence of co-pathology which was not well studied until fairly recently, and more data is needed in order to establish how diseases interact and influence each other and how this might be related to dysautonomia. Nevertheless, an easier issue would be to solve the dichotomy between clinical studies on dysautonomia that do not adequately provide proof of pathology and studies with histopathologic examination which do not consider ANS dysfunction symptoms. In this regard, we strongly recommend the use of the new NIA-AA Research Framework [27].

We are aware that these research criteria are difficult to apply in routine clinical practice, where it is more suitable to use criteria designed for a clinical diagnosis of AD, such as the 2011 NIA-AA Criteria [38], the DSM-V criteria, or others. Nevertheless, it is of lesser relevance in daily practice whether dysautonomia is caused by AD pathology, since both clinical studies and clinical practice use the same diagnostic criteria for AD. This extends to the signs and/or symptoms of cognitive impairment, autonomic dysfunction, and symptomatic treatments.

## 5. Dysautonomia as a Result of Comorbidities and Medication

AD is very common in the elderly, who frequently have associated comorbidities (cardiovascular disorders, diabetes mellitus) and use polymedication (antihypertensives, acetylcholinesterase inhibitors, antimuscarinic agents, antipsychotics, and antidepressants), which is likely to interfere with autonomic function (detailed in Table 1).

Cardiovascular disorders are very common in patients with AD. Up to 83% of these patients have arterial hypertension and 16% have ischemic heart disease, whereas 15% of them have suffered a cerebrovascular event [39]. Another study found a prevalence of 55.1% for arterial hypertension and 22.7% for cerebrovascular disease [40]. In a large study enrolling patients with different types of dementia and an episode of transient loss of consciousness suggestive of syncope in the last three months, hypertension was the most frequent comorbidity (74.5%), with a mean number of three antihypertensive drugs prescribed for each patient [41]. All the agents used in the treatment of hypertension could virtually induce or worsen orthostatic hypotension [42]. Among these, α and β-blockers, central sympatholytics, nitrates, diuretics, and combinations of angiotensin-converting enzyme inhibitors and diuretics or nitrates predispose one to orthostatic hypotension-related syncopal falls [41,43]. It is important to carefully prescribe them and monitor their use, since almost half of syncopal falls are related to orthostatic hypotension in patients with dementia [41]. It is noteworthy that these patients do not report the classical symptoms of orthostatic hypotension (e.g., dizziness) [44]. Instead, they have cognitive fluctuations, excessive sleepiness, slow falls without loss of consciousness, and fatigue, which might be misinterpreted as dementia symptoms, leading to unnecessary changes in antidemential treatment and delays in the proper management of orthostatic hypotension [44].

Another major comorbidity in the elderly is diabetes mellitus, with a prevalence of 9–25.7% [39,40]. Diabetes mellitus is regarded as a common cause of neuropathy-associated autonomic dysfunction. In non-insulin-dependent diabetic patients, parasympathetic dysfunction prevalence increases from 4.9% at baseline to 65% at 10 years follow-up, whilst sympathetic dysfunction rises from 6.8% at 5 years to 24.4% at 10 years follow-up [45]. Moreover, treatment-induced neuropathy (both oral hypoglycemic agents and insulin) has also been described [46].

Acetylcholinesterase inhibitors are the most commonly prescribed agents for cognitive symptoms in AD. There are conflicting results regarding the risk of orthostatic hypotension due to acetylcholinesterase inhibitor use. Many reports state that they do not increase the risk of orthostatic hypotension [47,48,49], whereas others have found a greater risk for syncope, bradycardia, and pacemaker insertion [50,51]. Another study states that donepezil is associated with bradycardia in doses that exceed 10 mg/day, which are no longer recommended [52].

Although an association between urinary incontinence and acetylcholinesterase inhibitor use has been hypothesized, it is rather linked to AD progression [53]. However, patients using acetylcholinesterase inhibitors are more likely to start anticholinergic treatment for urinary incontinence than those taking memantine [54], their concomitant use being common (9–10%) [55]. Antimuscarinic agents cause autonomic adverse events, namely dry mouth (29.6%), constipation, increased sweating, and urinary retention [56].

Neuropsychiatric symptoms commonly occur in AD, with up to 49% of these patients associating apathy, 42% depression, 40% anxiety, 39% aggression, 39% sleep disorder, 31% delusions, and 16% hallucinations [57]. Since most studies included were cross-sectional, the prevalence of neuropsychiatric symptoms is probably underestimated for patients with advanced disease [57]. A meta-analysis on the use of psychotropic drugs in patients with primary psychiatric disorders revealed that ANS dysfunction was common, but probably unrelated to medication [58]. It identified reduced heart rate variability only with the use of clozapine and tricyclic antidepressants, as opposed to selective serotonin reuptake inhibitors, serotonin–norepinephrine reuptake inhibitors, and other atypical antipsychotics (olanzapine, amisulpride, sertindole), whereas there were not enough data concerning benzodiazepines [58]. On the contrary, Hattori et al. concluded that quetiapine, olanzapine, risperidone, and aripiprazole could induce dysautonomia, mostly with quetiapine use [59]. Nevertheless, since antipsychotic use is related to cardiovascular events, careful monitoring is required [60].

## 6. Concluding Remarks

With increases in population ageing and growth, the prevalence of AD is expected to rise accordingly [61]. The proper management of AD implies addressing all the associated symptoms and conditions, in addition to the cognitive and psychiatric aspects [12]. Among these, ANS dysfunction is particularly important, provided that it has prognostic relevance for morbidity and mortality [4,12]. Moreover, it has been suggested that dysautonomia might accelerate cognitive decline [13], and some authors even regard it as an early biomarker of neurodegeneration [12]. Since ANS dysfunction is a dynamic process that perpetuates throughout the course of AD, we advocate frequent thorough assessment, even in asymptomatic patients, and the employment of specific corrective measures [4,12]. In this review, we propose four potential causes for ANS dysfunction in patients with AD (i.e., AD pathology, brain co-pathologies, comorbidities, and medication), but further studies are required in order to confirm these links and find related interventions that could improve the ANS burden in AD.

## Figures and Tables

**Table 1:** Common drugs used in patients with Alzheimer's disease (AD) that induce autonomic nervous system (ANS) dysfunction.

Antihypertensives	α-Blockers, β-Blockers, Central Sympatholytics, Nitrates, Diuretics	Bradycardia, Syncope, Orthostatic Hypotension
**Acetylcholinesterase Inhibitors**	donepezil, galantamine, rivastigmine	bradycardia, syncope, orthostatic hypotension
**Antimuscarinic Agents**	darifenacin, propoverine, solifenacin, tolderodine	dry mouth, constipation
	trospium	constipation
	oxybutynin	dry mouth, urinary retention
**Typical Antipsychotics**	haloperidol	cardiovascular events, sexual dysfunction
	chlorpromazine	cardiovascular events, orthostatic hypotension, dry mouth, constipation, urinary retention
	thioridazine	orthostatic hypotension, dry mouth, constipation, urinary retention
**Atypical Antipsychotics**	quetiapine, clozapine, olanzapine, risperidone, aripiprazole	cardiovascular events, dry mouth, constipation, urinary retention, sexual dysfunction
**Antidepressants**	tricyclic antidepressants	cardiovascular events, dry mouth, constipation, urinary retention
	selective serotonin and serotonin-norepinephrine reuptake inhibitors	dry mouth, constipation, diarrhea, sexual dysfunction, excessive sweating
